# Half-Yearly Report of the Progress of Medicine, from January to June, 1812

**Published:** 1812-07

**Authors:** 


THE
### Medical and Physical Journal.

1 OF VOL. XXVIII.] JULY, 1812. [NO. 161.

*For the Medical and Physical Journal.*

### HALF-YEARLY REPORT *of the* PROGRESS *of* MEDICINE, *from* JANUARY *to* JUNE, 1812.

(No. VI.)

"It is among the first duties of those who conduct a work that has obtained an extensive circulation, to diffuse a knowledge of every thing that may be serviceable to mankind; and to consider the amusement of their readers as a very subordinate object to the communication of useful intelligence."—EDIN. REV.

IN the efforts which have hitherto been made in this Report, to present to the medical public a condensed view of the progress of the science, within stated intervals, utility has been the fundamental principle by which those efforts have been directed; and, though the writer may not be entirely inattentive to the composition, he has been flattered by the approbation of the profession into a belief, that, in the main object, usefulness, he has been moderately successful. The incentive afforded by the approval of his brethren, joined to the consciousness of intending to act with fairness, will impel him to additional exertions; in which he has a hope to diffuse a knowledge of facts that may be serviceable to the profession at large, without being offensive to individuals; and, according to the precept of our immortal bard, "nothing extenuate, nor set down ought in malice."

To observe even but a step onward to that point of perfection to which we would believe every thing is finally destined, is a satisfactory counterpoise to the retrograde movements to which all human knowledge is at times doomed. Marking this progression, we are enabled to calculate, that, in the aggregate, science, in all its departments, is on the advance. In particular branches of the medical art, perhaps in all, this fact, we believe, is quite established. Chemical accuracy in the composition of remedies, simplicity of prescription, mechanical dexterity of operation, and the the development of causes in many instances amounting to a demonstration, shew the present period to be that in which the science of medicine has become more perfect than in all preceding times.

This is peculiarly obvious in the minute knowledge of the structure, and the proportionate acquaintance with the functions, of all animated creatures, which distinguish the anatomists of the present day.

An inquiry into the precise alteration that parts have undergone by the action of disease, as ascertained by examination after death, under the denomination of MORBID ANATOMY, connected with the previous symptoms, which has, since the publication of Dr. Baillie's work, been particularly attended to in this country, gives an assurance of the acquisition of a still more exact knowledge of organic diseases, or those morbid effects, which depend on altered structure for their cause. The influence that organic alterations have in producing extraordinary and obscure sympathies, the effects of which are often seen in distant parts, may possibly be demonstrated by this attention to morbid anatomy; nor have competent judges of the subject been without expectation that, even the natural structure of minute parts may become better understood by these investigations.

The present period has presented us with a very favorable specimen of this species of inquiry, in an extensive volume upon the *Morbid Anatomy* of the first passages. Dr. Monro, the Professor of Anatomy, Surgery, and Medicine, at the university of Edinburgh, has done a double service to the science by the publication of this volume, by the specific information which his work contains, and by the example which it affords; and which we trust will be followed by a similar inquiry into the altered structure of other parts of the frame, and its consequent results. The morbid anatomy of the brain, the heart, the lungs, the liver, the urinary organs, and the organs of generation, present, each of them, perhaps, a subject for a volume. The morbid condition of the hepatic system, in the direct or in the indirect operation it has on every part of the frame, presents an inquiry of peculiar interest. The nervous feelings, as they are vaguely

 called, called, the irregularity of the actions of the chylopoetic viscera, the languors, the irritability, and a long train of strange sympathies which occur in, and seem to depend on, some alterations in the liver, it must be confessed are yet imperfectly understood. To designate all these morbid feelings by the same term *bilious*, meaning a redundant secretion, seems as disgraceful to common sense as to science. Those persons which, in this idle phraseology, are called bilious, are presumed always to suffer by a superabundant secretion of that fluid, when the rational probability is, that, in a morbid state of the liver, there is a deficient secretion of bile in very numerous instances; and that this deficient secretion is the source of those sensations that make life a burthen.

The volume which has given rise to these cursory remarks, contains near 600 pages, and twenty-one plates. It professes to treat only of the diseases that occur in the course of the passage that begins at the mouth and terminates with the rectum. The œsophagus, the stomach, and the canal of the intestines, are of course its proper subject. The morbid states of the œsophagus here treated of are inflammation, ulceration, gangrene, polypi, spasm or stricture, paralysis, scirrhus, and cancer. The stomach and intestinal canal are treated of under all the above circumstances.

The obstructions that occur in the intestines from mechanical means, as extraneous bodies impacted in the canal, are also particularly noticed; and, on the subject of *alvine concretions*, there is a section of peculiar merit, illustrated by neatly engraved, and probably correct, plates. The effects of arsenic, opium, and mineral acids, are treated of, with the means of obviating the effects of poisons; but in a way to excite a wish that the subject may be again taken up, rather than to satisfy the mind.

In the particular details we are induced to select, section xviii, p. 217, which inquires into the history, effects, and results, of "*the deposition of albuminous matter in the cellular coat of the alimentary canal.*" The originality of this section will apologise for making the quotation.

"This organic derangement is of a very peculiar nature, and and very generally affects at the same time different bowels of the abdomen and pelvis, and also the lymphatic glands in the vicinity, &c. &c.*

"There is some variety as to the color and quantity of the albuminous matter, which has been deposited; generally it bears a strong resemblance in color and consistence to the white part of the skin of an orange, but it is not perfectly homogeneous as to its texture, being in some cases somewhat harder and more transparent than in others; and I have seen it," says the author, "precisely of the color and consistence of equal parts of bees'-wax and tallow. Upon putting the albuminous matter into an acid, or into alcohol, it becomes considerably harder."

To this short history of the substance itself, is subjoined a general detail of its effects on the villous coat, (it is in itself stated to be a disease of the cellular coat) on the lymphatic glands, the liver, uterus, and testes.

Dr. Monro received from Dr. Barclay a uterus which had been the prey of this disease: it was as large as the gravid uterus at the full time, and its parietes were an inch thick. It was lined with a layer of coagulable lymph, and there were several irregular shaped masses of a white color within it, of different sizes.

The organic alterations which this disease produces in the liver, Dr. Monro has had many opportunities of observing. "It appears," he says, "on the surface of the liver in the form of small round tumors, which in color and consistence resemble pressed curd. The tumors have a circumscribed margin, flattened apex, are of different sizes, rarely larger than a walnut." The liver does not retain its usual figure, but becomes much thicker at the edges; and increases in weight to 20 lbs. avoirdupoise. The increase in size of the liver does not bear, however, a regular proportion to the increase in weight; for, this albuminous matter being much heavier than the parenchyma of the liver, the volume of the viscus is not enlarged to a degree that might be expected.

---

* The connecting passages having been inserted in an Analysis of this work, printed in our last volume, p. 509, we refer our readers to that place in preference to a re-insertion.

The

The peritoneal coat that covers these tumors, is found to be of an unnatural thickness, and the tumors themselves are found of different sizes throughout the substance of the liver; a fluid like the coagulable lymph of the blood being sometimes lodged in their centre.

When macerated in water, these tumors become soft and flabby; and, when examined by the naked eye, often appear perforated; but blood-vessels have not been observed within their substance.

When albuminous matter has been deposited within a part of the substance of the liver, the Professor has generally remarked, that another part of the same liver was of the consistence of jelly; and this change into a gelatinous form, is conjectured to be preparatory to its absorption.

The substance of the liver between the albuminous tumors, is of different shades of yellow or green; and the tumors themselves, in some cases, completely obstructed the biliary ducts. In two cases of this kind, the contents of the gall-bladder were analysed by Dr. Duncan, jun. and found to possess qualities very different from bile. A dram of this fluid contained only four grains of matter like the white of an egg, and so small a quantity of the yellow resinous material, as to be imperceptible in the nicest balance.

The importance of this morbid alteration in the structure of the liver, from which resulted the total change in its actions, and the almost annihilation of its secreting functions, have induced us to dwell more particularly on it; and especially as it seems to have been but little attended to before. We are not able yet, perhaps, to give the pathognomonic symptoms of this disease. These symptoms, as detailed by the author, are probably not peculiar to this specific alteration in the hepatic structure. Indigestion, acidity, nausea, pain at the top of the shoulder, are the common effects of a morbid state of the liver; and jaundice and ascites, its usual results, whether that morbid state is this deposition of albuminous matter in its substance, or any other change. This is said, however, subject to correction; and we should be glad to find that the pain in the right shoulder, accompanying very frequently quently a diseased condition of the liver, is peculiar to, and characteristic of, the albuminous tumor of that viscus.

As an ample analysis of this, in many respects valuable, volume, has been inserted in a preceding Journal, (vol. xxvii. p. 494,) we shall not pursue the observations on its contents any further; but attend to the discoveries and labors of an indefatigable surgeon, of high eminence, and extensive means of acquiring a knowledge of diseases, especially those of the urinary organs.

As long since as 1806, Mr. Home announced his discovery of the existence of a "*Middle Lobe of the Prostate Gland.*" In the month of February of that year, this discovery was laid before the Royal Society of London, in a paper descriptive of the fact itself, detailing its peculiarities, and substantiating the author's claim to the discovery. In the volume before us, "Practical Observations on the Treatment of the Diseases of the Prostate Gland," this paper is republished as an introductory chapter to the practical part of the work; and now, being for the first time directly submitted to the profession, comes to be noticed as a discovery of the present period.

In a report professedly historical, it is not required that a critical examination of the fact or its probable consequences should be entered on, or the views and objects of the discoverer investigated; a history of the occurrence, unconnected with the opinions of others, separated from doubts of the real existence of a middle lobe of the prostate gland, is all that will be here attempted.

In the month of December 1805, Mr. Home's attention was excited to this subject by circumstances not necessary here to be related. An anatomical examination under Mr. Brodie, of whose skill in such researches no doubt can exist, was instituted. Many subjects were examined, and in a healthy one, twenty-five years of age, the natural appearances of this part were distinctly marked. These appearances are thus described:

"On turning off the vasa differentia, and vesiculæ seminales, exactly in the middle of the sulcus, between the two lateral lateral portions of the prostate gland, there was a rounded prominent body, the base of which adhered to the coats of the bladder. It was imbedded not only between the vasa deferentia and bladder, since they were in part spread over it, so as to prevent its circumference being seen, and they adhered so closely, as to require dissection to remove them; nor could this be done beyond a certain extent, after which the same substance was continued from one to the other. This proved to be a lobe of the prostate gland; its middle had a rounded form, united to the gland at the base next the bladder, but rendered a separate lobe by two fissures on its opposite surface. Its ducts passed directly through the coats of the bladder, on which it lay, and opened immediately behind the verumontanum. By means of this lobe, a circular aperture is formed in the prostate gland, which gives passage to the vasa deferentia."

Many other subjects were examined, with the view of substantiating this discovery: in some this middle lobe was more obscure, in others larger and still more obvious, than in the above history. In one subject, twenty-four years of age, "it was found still larger and more distinct." A representation of this is given in a plate.

To this plain statement of an historical fact, we will annex the author's reflections on its importance, and on the consequences of this middle lobe getting into a morbid state.

"This newly-acquired anatomical fact enables us very clearly to understand the nature of a disease, which it was not possible to have a correct idea of, when we were ignorant of the existence of the part in which it takes place. It not only explains the situation of the tumor, the apparent want of connection with the body of the gland, and the narrowness of the base, which in many instances is met with, but it solves what has ever appeared to me," says Mr. Home, "the greatest difficulty, how it should protrude into the cavity of the bladder. This arises from the hard substance of the coats of the vasa differentia being in close contact, and bound down upon this lobe; so that, from the first enlargement, it must immediately press upon the inner membrane of the bladder, which can make very little resistance."

This

This inquiry is pursued at large in Mr. Home's volume, and the nature, symptoms, consequences, and management, of the diseased state of the middle lobe of the prostate, under various points of view, minutely investigated. But these, as expressly belonging to the analytical part of our work, will be reserved for a future period; when it will receive a full exposition, with the confirmations of, and objections to, the fact and opinions, in conjunction with Mr. Foot's "Review" of Mr. Home's statements and claims.

Intimately connected with this branch of our Report is the valuable work of Mr. Travers, in which inquiries into the "Process of Nature in repairing Injuries of the Intestines, illustrating the Treatment of penetrating Wounds and strangulated Hernia," are made with a degree of acute precision, pursued through a long course of experiments, that is highly creditable to their author, and promising great advantages to the practice of surgery.

The object of Mr. Travers being to ascertain, precisely, what takes place within the abdominal cavity after the infliction of wounds penetrating that cavity and entering some portion of the intestinal canal; he adopted a course of experiments, which, had it been possible to have arrived at his results without, we should certainly have reprobated. Wounds were inflicted on the intestines of dogs: at certain succeeding periods, the dogs were destroyed, careful dissections were employed, and the actual state of the natural process was manifested. It is obvious, that by this course of inquiry, facts of great importance must be arrived at.

Among them we can only notice, that the cavity of the abdomen, as it is called, in a natural state is always full; or that the contained parts lie every where in contact with the parieties and with each other. The intention of Nature in this seems plainly to be, to afford a counterpoise against the consequences of wounds of this part of the frame. When in wounds of the intestines the fœcal contents escape into the cavity, death is the inevitable consequence: but it will be seen, that, if the wounded part must, from the natural state of the abdominal cavity, fall into contact with some neighbouring part, and that the wound must also be closed up by the the contact of these parts, but little chance is left for the escape of the contents of the intestines; and, the adhesive inflammatory process going on, the parts are aglutinated. 2. That it is essential to keep the patient under wounded intestine, in a state as nearly as possible of abstinence, and this is strikingly illustrated by experiments. 3. That all attempts made ab extra to prevent or to close an artificial anus, are futile or injurious, perhaps fatal, until the passage per anum is first restored. As this is a practical fact of great importance, we may perhaps stand excused for quoting the passage in which it is enforced, although it has already appeared in our former volume.

"I need scarcely say (observes Mr. Travers) that, in substituting the natural for the artificial anus, it is necessary to proceed with the most delicate caution. It is common for the more solid parts of the food to lodge and be detained in the renovated portion of the canal. There is a tendency to accumulation wherever an extensive wound has been inflicted upon the intestine: first, from the contraction proportioned to its extent, which has been shewn to take place at the moment of the injury: secondly, from the contraction which accompanies the healing process: thirdly, from the impaired action of that part of the gut which has been the subject of the lesion. The discharge per anum should therefore be attentively observed, both as to its consistence and quality. If it cease, or ceases to correspond with the diminished evacuation from the wound, under the continued use of injection, the belly will become tender, and the febrile symptoms reexcited. Under such circumstances, the exhibition of mild laxatives by the mouth may prove advantageous; but, should the constipation be obstinate, and the abdominal pain and tension continue, the wound must without hesitation be dilated, and the bowel be again unloaded."

It cannot be too strongly impressed, that the fœcès resume their natural channel not because the wound closes, but the wound closes because the natural passage is restored. It is a maxim of the utmost importance in the treatment of these injuries, that evacuation from the intestinal canal is one of the principal means of obviating or removing the symptoms of inflammation: and, if this cannot be effected per anum, it must by the wound.

Among other remarkable and valuable facts contained in this volume, we cannot refuse to notice the manner in which the ligature employed in wounds of the intestines is disposed of, after the purpose for which it is employed is effected. Dr. Thompson had ascertained, by experiments, that when a wound of the intestine was secured by ligature on the cut edges, the ligature, during the healing process, passed inwardly, and was deposited in the canal of the intestine: and by this mode Nature provided for its expulsion. It was not, however, explained how this was effected, though the fact was certain, until the publication of Mr. Travers' work. From the elucidation of the process by this ingenious surgeon, it is become evident that the ligature will, by necessity, fall into the canal of the intestine, when liberated by ulceration. We should do an injustice to the explanation of this interesting fact, did we not give it in the perspicuous language of the author.

"The inflammation which a ligature induces on either side of it, is terminated by the deposition of a coat of lymph exterior to the ligature, which quickly becomes organised. When the ligature thus inclosed is liberated by the ulcerative process, it falls of necessity into the canal, and passes off with its contents. Ligatures of every description (unconfined at the external wound of the abdominal parieties) separate into the canal; not from any law in the economy, but from their speedy and complete investment (as just stated) by the uniting medium."

There is not, we believe, a more striking instance in the animal economy, of the consummate wisdom with which the whole is constructed, than is evidenced in this provision for the removal of a foreign body, the application of which is essential to the cure, as is its subsequent removal. And the fact is still more impressive, as the process by which the ligature is removed is excited by the ligature itself, and would have no existence were the ligature not there to be expelled.

The phenomena, history, and treatment, of mortified intestinal Hernia, occupies, with much propriety, a considerable able portion of Mr. Travers' work. The following is a short and very inadequate summary of this interesting subject.

When hernia terminates fatally, it commonly does so from inflammation spread along the intestine.

The first point of the inquiry is intended to ascertain, whether this diffused inflammation, when spread over the intestine, arises merely from a portion being involved in a stricture, or from the stoppage of the passage of the fœces, which is generally attendant on the strictured state of the intestine.

The second inquires what the powers are which Nature employs to remedy an intestine sphacelated by hernia, and the peculiarities of her process.

In the third, an application of the facts and principles, developed in this inquiry, is made directly to practice.

The practical conclusion is, that in cases of strangulated hernia there are always two things to be kept in view: one, that the stricture shall be removed; the other, that the obstruction in the passage of the intestine, arising indirectly from the stricture, shall also be removed. The first is fully effected by the common operation; the second, which if not removed will be fatal, must be treated with purgatives and injections. But, if purgatives and injections do not remove this obstruction, the question, "*what is then to be done?*" is forcibly put.

As it seems, and actually is, essential to remove the substances collected in the intestinal tube, and which are necessarily keeping up an increasing distention, a distention which if not removed must be fatal, it becomes a point of momentous consideration, whether the accumulation should not be evacuated by the lancet, through a strictly artificial anus, the intestine being already fixed by adhesions to the parieties.

We repeat that this is a momentous consideration, and, without Mr. Travers positively advising it, we feel it a duty to call the attention of surgeons to the suggestion.

In this division of the science, it is necessary to notice further a very useful publication by Mr. Allan Burns, on the surgical Anatomy of the Head and Neck, illustrated by engravings; a Description of the Arteries, by Dr. John Barclay; and Plates of the Brain, by Dr. Alexander Ramsay, a teacher of anatomy at Edinburgh.

It has been a frequent, and we lament a just, remark, that however correct, however scientific, the English anatomists are, as teachers, that when they appear before the public as authors, they are often careless in arrangement, and slovenly in composition. But, of the engravings which accompany, and are intended to illustrate, their works, we are compelled to speak even less favorably. While on the continent, under every depression that can result from insecurity, tyranny, slavery, and poverty, elaborate works on science and art issue from the press, ornamented with engravings of exquisite execution; in this country we see such works encumbered with plates of the meanest workmanship, made after designs, in every particular of composition and drawing, disgraceful to the arts. It is remarkable that these defects have existed in the most glaring manner in those works intended to illustrate the *sensorium commune* and general distribution of the nerves. If, a year or two since, we saw, in London, a gentleman, in the eagerness and ardor of youth, submit himself to censure for publishing a volume on this difficult, though peculiarly interesting, subject; Dr. Ramsay's plates of the brain have left no triumph to the metropolis of Scotland.

It is not meant to be implied, however, that Dr. Ramsay is not competent to something much better than these plates of the brain. We believe he is, and shall have a sincere gratification to hail him as the author of a work that may be placed, without hazard of depression, by the side of Sommering. Cannot some species of feeling impel our countrymen to emulate the continental authors upon the interesting subject of the brain and nervous system? Why are we thus left at a distance behind? It is not a conjecture that either physical or commercial means are wanting. Why then do we see only this trifling and buzzing about the subject? this eternal manufacturing of books for tyros and boys?

From these details upon structure and its morbid alterations, tions, we turn to a less certain, though, to the imagination, more grateful, branch of inquiry—the physiology of sentient beings.

Dr. Buchan, in a little, almost aphoristic, volume, under the title of *Bionomia*, has concentrated opinions concerning life and health, or the laws by which these states of existence are governed. It is intended as introductory to a course of lectures on a subject of acknowledged importance, and calling forth the highest powers of the human mind. We are not, however, assured that the author does actually purpose such a course; though no hesitation can remain upon the propriety of associating an extended and erudite view of the philosophy of vitality, and an investigation of the laws of life, with the labors that profess to go no further than simple elements, and teach only the structure and mechanism of the animal frame.

"At a time when philosophy appears to be almost totally immersed in the consideration of matter, and its various modifications, it will be deemed no improper attempt to call some share of the public attention to the study of that class of natural phenomena which are connected with the influence of the principle of LIFE."

To this observation there will not be a dissentient voice; and if our approval can in any degree influence Dr. Buchan to elucidate, in a course of public lecture, the *Physiology of Sentient Beings*, and rescue the human mind from the almost exclusive contemplation of inanimate matter, he has it most fully.

Though chemistry, the direct object of which is matter, or some of its modifications, independent of, or unconnected with, the vital principle, now occupies so large a portion of the time and talents of mankind, it has not, nor ever can it, assume an exclusive authority over human pursuits. But, if its operations are necessarily confined to dead matter, they are often made with a view to the effects produced by this matter upon the functions and vital principle of animals. The process of respiration has been explained by this science; and a variety of attempts have been made by the chemist to eulcidate elucidate the *modus operandi* of substances, either as poisons, remedies, or nutriments.

With relation to the constituent principles of bodies, their combinations with and effects on each other, the science of chemistry has been *most* successful; but it does not appear that this success has been extended far, if at all, to the illustration of their actions upon the functions of life, or that we are made better acquainted with the operation of nutriments, poisons, or medicines, by the investigations of the laboratory. In the composition of remedies, and in the avoidance of incongruous and decomposing combinations, we acknowledge valuable instruction from the chemist; but in the application of substances, to influence the actions or functions of animated nature, whether in health or disease, we do not perceive one ray of light to have emanated from this science. Dead matter is its proper object; the vital principle has not yet come within its grasp.

In the experimental inquiry, noticed in the preceding Report as having been instituted by Mr. Brodie, it was not obvious that chemistry afforded a single illustration; on the contrary, the happiest elucidation that the science has, as regards life, received a shock that renders the truth of its principle doubtful. This gentleman is still pursuing the investigation; and, as the objects of it are the mineral poisons, arsenic, muriate of barytes, tartarised antimony, and oxymurias of mercury, we may perhaps, when the details come before the public, see that chemistry will afford explanations consonant to its own principles, and directly applicable to vitality.

Inefficient as this science yet appears to be when applied to the doctrines of life, it is, like the mathematics, all powerful within its proper sphere, and upon those subjects to which it seems to have been destined by the law of nature. To one of the most potent and active material agents of this globe, heat or caloric, it is peculiarly applicable. The operations and actions of caloric extend, indeed, to the vital principle; but its chemical *modus operandi* on this principle remains in darkness. The promised "View of the Facts ascertained concerning Heat," by Professor Leslie, will, perhaps, perhaps, illuminate this obscurity, and enable us to go with more success into this subject, in our next Report.

Upon the Materia Medica, Botany, and Natural History, the present interval does not present much novelty of interest. The attempted appropriation of an American plant of the genus *Eupatorium,* as the active material of the Eau Medicinale, though it has failed as to the discovery of the substance that affords the evacuant property to that nostrum, has the merit of calling the attention of the faculty to a family of very active plants. This conjectured basis of the Eau Medicinale, the *Eupatorium perfoliatum* of North America, possesses active qualities; and a paper by Mr. Pursh, in the preceding volume of this Journal, relates some pointed instances of its successful employment as an evacuant in the Lake Fever of that country. It seems to have been among the earliest of the North-American plants brought to our gardens; and, as it is destitute of beauty, it must have been imported for its reputation as a remedy. The only species of this genus indigenous to England is the *Eupatorium cannabinum,* a plant possessing medicinal properties of a quality to claim some attention from the practitioner.

The physiology of vegetables has received some elucidation in a pleasing Essay on the Probability of Sensation in Plants, by Mr. Tupper;* and natural history has attracted to it an increased attention by a republication of Buffon, with additions and illustrations, by Mr. Wood. This last department of science has also received unquestionable benefit by the opening of Bullock's Museum, rendered more interesting by the addition of numerous specimens, and an improved arrangement.

The theory and practice of medicine has obtained illustration and improvement on some important diseases by the

---

* In one of our preceding Numbers (vol. xxvii. p. 417.) we have given an analysis of Mr. Tupper's Essay, for which we were indebted to a contemporary Journal. A re-perusal of the Essay itself, does not induce us to retract the opinion there given. It is a collection of curious facts, on a subject of much interest, and tending to elucidate the varied forms that the principle of life is subjected to, and the analogies that exist among these forms.

publications publications of Cheyne, Gillman, Syer, Saunders, Armstrong, and White; and by the second volume of the Transactions of the Medico-Chirurgical Society of London.

The importance of inquiries into the properties of the rabid virus, and into the quality of the symptoms arising in the course of its action upon animal systems, not founded on hypothetical argument and vague theory, but taken from direct observation of the phenomena, will be readily acknowledged. And it is this precisely that gives a degree of value to Mr. Gillman's Dissertation. That gentleman closely observed the progress of the disease, particularly in quadrupeds; and, after it had, in his cases, as in all other instances we believe, terminated in death, he examined the bodies, or caused them to be examined by persons highly competent to that office: yet these inquiries have not suggested any method of cure. But the state of the stomach seems to point to a principle, which may hereafter, if confirmed into a law by subsequent observations, bring us more acquainted with some phenomena of the disease.

In some instances the stomach was found to have a sort of pustular appearance, approaching to sphacelus; in others, this organ was perfectly free from such appearance. In those animals where this pustular state of the stomach occurred, there was but little of violent convulsion; they presently sunk into a state of calm debility, in which they died. In those animals where the sphacelated state of the stomach was absent, the convulsions were violent, the struggles enormous, and every active symptom aggravated until the system was worn out. If we apprehend this aright, it will establish, suggest at least, two varieties of this disease: one accompanied with sphacelus of the stomach, indicated by torpor and debility, requiring in its progress powerful stimulants conjoined with tonics; the other, without any detectable organic alteration, attended with active convulsions, and excessive action of the system, requiring bleeding, general evacuation, and sedatives. But so obscure still is this disease, that this inference must be considered as, in a great degree, conjectural, and to be confirmed or rejected by future observations.

We possess no remedy for Rabies; all our efforts, there-

 fore, fore, must be directed to the prophylaxis: and for the conduct of this Mr. Gillman lays down rules, which, as far as they go, ought never to be departed from. Since Mr. Gillman's publication appeared, a man has died of rabies in the neighbourhood of London, in whose stomach appearances existed precisely similar to those in the stomach of the dog, as they are represented in the plate attached to this Dissertation. We have not learned what were the symptoms in this case, and therefore do not know if they accord with the above inference.

The prevalence of the strumous diathesis in this island, manifesting itself in an infinite variety of forms, from tumid glands in the neck to the severest cases of tuberculous consumption, has almost been a reproach to our country, and certainly a stumbling block to our physicians. Under these circumstances of frequency of occurrence, and difficulty of management, it is not surprising that almost the whole of the materia medica has been pressed into the service against scrofula. Dr. Armstrong has now added to the list the *Ammoniæ Carbonas.*

This remedy, in his hands, appears to have been efficacious in a remarkable degree. Several cases are related by him; the result of which encourages a hope, that in the carbonate of ammonia may be found a remedy decidedly advantageous against the strumous diathesis.

It was given by Dr. Armstrong in the following form: ℞. Infus. Gentian. Comp. ℥vijſs. Ammon. Carbon. ʒſs. Tinc. Gentian. Comp. ℥ſs. Misce. Capiat Cochlearia tria ter die. This formula was employed for children, varying the dose from one table spoonful to four, three times a day, according to circumstances. When the patients were adults, the carbonas was increased to two scruples or a drachm, in the eight ounce mixture. During the employment of this remedy, there was an occasional interposition of calomel purges.

Of the practical Essay on the Diseases of the Rectum, by Mr. White, an analysis has already appeared in the preceding volume of this Journal. We have little to add to that analysis; but the subject should not be dismissed without

 observing observing that publications, like Mr. White's, containing opinions illustrated and supported by authentic cases, at the same time that they add to the stock of medical facts, directly tend to meliorate the science.

A Treatise on the Management of Infants, by John Syer, surgeon, has attracted our notice as a copious commentary on a subject which has been too little attended to, though of great importance, and calculated to excite the feelings in no common degree. The object of the writer is to combine popular instruction with medical practice, an incongruous mixture often attempted, and always uselessly, except when it shews distinctly, that, in the administration of remedies, and the management of diseases, a species of skill, the result of education, habit, and experience, combined with observation and reflection, is always requisite to success.

The volume comprises a consideration of the remote causes of the derangement of the health of infants, with a view to the prevention of disease; and the history and treatment of individual diseases restricted to the term of childhood.

The author attempts to support his notion of conveying popular instruction on a subject of peculiar difficulty, and which, if any art does, requires a rigid course of preliminary studies. "Although (he says) the difficulties in many parts of adapting medical instruction to the comprehension of the general reader are too obvious to be concealed, yet the attempt to reduce medical precepts to a certain share of popularity, should not be treated as wholly chimerical; for the same objection will apply, with more or less force, to popular treatises on the general principles of law, agriculture, or astronomy, of which the elements at least may be expected with facility and advantage to be acquired."

We must observe on this, that, in medicine, the popular diffusion of *precept* does not, as in law, agriculture, or astronomy, end in furnishing materials for conversation, but in a practice necessarily crude, uncertain, hazardous always, sometimes fatal. No man conducts a law suit or constructs a deed on the knowledge he gets from a popular treatise; neither does he undertake to cultivate his fields, calculate eclipses, or navigate a ship, on the precepts he finds in such a volume. But, in the affair of health, of life, he rashly acts on the presumed knowledge, acquired, he believes, where knowledge, such only as should enable him to prescribe, was never yet acquired. A precept or aphorism, is a condensed form of human knowledge, the result of a perfect acquaintance with elementary principles, extensive views, and profound information, on all the bearings and parts of science. Those who would act on aphoristic *dicta* with success, must bring to them a certain portion of the learning that furnished them; a habit of examining diseases, and practical dexterity in the application of remedies. A taylor does not cut out a coat by precept, nor does the mechanic work by aphorism—yet, in medicine, give the precept, and practice follows.

A medical work, if at all valuable, is so to the profession: to the people, when intended to be popular, it is worse than useless, because, if they read it, it invariably induces them to prescribe without enabling them to discriminate diseases, or to understand the properties and powers of medicines.

These observations are applied to Mr. Syer's volume only as far as it is intended to be a popular treatise ; if it should induce mothers to call in the assistance of the author, instead of prescribing themselves, it may answer a good purpose.

The death of Mr. Saunders,* in February, 1810, deprived the

* John Cunningham Saunders was born at Lovistone, in Devonshire, on the 10th of October, 1773. He received the rudiments of education at Tavistock. In 1790 he was placed with Mr. John Hill, surgeon, of Barnstaple; with whom he remained five years, and then removed to London to complete his medical education. He became a pupil at St. Thomas's and Guy's Hospitals: at the end of two years he was appointed demonstrator of anatomy at St. Thomas's. It is sufficient evidence of his merit, that, on the appointment of Mr. Astley Cooper to the anatomical chair, he was elected to the office which that celebrated surgeon had recently filled. With the short interval of the summer of 1801, he continued in the office of demonstrator of anatomy, until the winter in which he died. In 1804 he published a proposal of founding a charitable institution for the cure of the diseases of the eye and ear. This proposal was carried into effect, and the institution now (1812) bears the name of the LONDON INFIRMARY FOR CURING THE

 DISEASES

the profession of an acute, enterprising, and scientific, practitioner. Having directed the powers of a mind of uncommon force, with an extraordinary degree of application, to a particular department of the medical art, the progress he made toward perfecting that department, has secured to him a probable permanence of reputation.

We had occasion formerly to observe, that, not many years since the practice of the oculist was chiefly confined to foreigners. A baron, a chevalier, or a sieur, threw over this highly interesting branch of medical science a charlatanic coloring that almost forbade the regular surgeon to touch it. This prejudice has, however, been overcome, and medical practitioners of character now undertake the management of diseases of the eye. The gentleman whose labors on this subject have suggested these observations, has considerably advanced our knowledge of the morbid states of the organ of vision, and improved the practice to a degree that claims commendation. But it is particularly the object of this Report to notice a posthumous work of Mr. Saunders, published by his friend and colleague Dr. Farre, with the title of a "Treatise on some practical Points relating to Diseases of the Eye." Of this we have given a full analysis in our twenty-seventh volume. We have now, therefore, only to call the attention of our readers to it as containing many

---

DISEASES OF THE EYE. Why it has been limited to diseases of the eye, and that of the ear, according to the first proposal, rejected, we are not informed. Besides the advantages that accrued to the art, and the benefit derived to patients by the skill and manual dexterity of Mr. Saunders, he added to the character of the medical literature of this country, by the publication of a folio volume, with plates, on the Anatomy and Diseases of the Human Ear (vide Med. and Phys. Journal, vol. xviii. p. 5), and an Essay on the Inflammation of the Iris, as a specimen of a series which he meant to communicate on the diseases of the eye. While thus prosecuting with ardor and effect the improvement of the science to which he was devoted, his labors were arrested by the hand of death. A disease of the brain, which had gradually been stealing upon him, terminated his existence by a stroke of apoplexy, on the 9th of February, 1810. We are willing to suppose some future biographer of British medicine may thank us for these short notices: for an ampler detail we refer to Dr. Farre, in the posthumous work noticed in the text.

valuable valuable observations and suggestions, with ingenious and effectual modes of practice—on inflammation of the conjunctiva in infants—on inflammation of the iris, and the influence of the extract of belladona to prevent the consequent obliteration of the pupil*—on the cure of the inversion of the upper eye-lid by excision of the tarsus—on some of the more important terminations of ophthalmia, by effusion of lymph, by suppuration, by slough, by ulceration, by protrusion of the iris—on some of the more important changes of structure in the eye—on the congenital cataract.

The momentous genus of diseases, one species of which robbed society of the talents that had so early been displayed in Mr. Saunders, has received an ample investigation by Dr. Cheyne, in an Essay on Apoplexy and Lethargy. This ingenious pathologist has given, with much precision, the history and the anatomy of apoplexy, as he denominates the examination, *post mortem*, by which the morbid appearances, taking place in the brain and other organs, are demonstrated. The hints we have given, in the first part of this Report, respecting the influence that morbid states of the *liver* have on all parts of the system, seem to receive some confirmation by facts noticed in this essay. Sometimes as the cause, and sometimes as the effect, possibly, of apoplexy, the hepatic system has been found considerably changed from the healthy state. The observations of Dr. Cheyne on this subject may lead to a fuller inquiry into the reciprocity of actions that take place between the liver, the corporeal functions, and the mental principle. The effect of this essay must be to make us better acquainted with the morbid alterations of structure taking place in attacks of apoplexy, the phenomena of the disease, the methods of treatment, and the distinctions existing between it and lethargy.

The publication of a second volume of the Transactions of the Medical and Chirurgical Society, shews very distinctly the flourishing state of that body, by the number of articles which it contains, the character of the contributors, and the

* This effect of the belladona was first given to the public by Mr. Saunders in this Journal, vol. xvi. p. 55.

additions to its members. But, as it is intended to give an analysis of this volume at a future time, nothing more than its more prominent features will now be noticed.

In the medical department will be found a case of hydrocephalus internus; a paper on the use of oil of turpentine in tænia; a case of secondary small-pox, to which is appended an historical detail of similar cases, collected from the printed records of medicine; an account of a severe case of erythema, unconnected with mercurial action; on painful affections of the side with tumid spleen; case of recovery from the effects of a large dose of arsenic; and on the effects of arsenic as a remedy for the bite of venomous snakes. The chirurgical class will be considered as particularly valuable when it is seen that Mr. Astley Cooper, Mr. Travers, Mr. Chevalier, and Dr. Hutchinson, have been contributors. The value of the chemical part is sufficiently characterised by the paper of Dr. Henry on the urine discharged in diabetes mellitus, by Dr. Bostock's experiments and observations on the serum of the blood, and by Dr. Marcet's chemical account of various dropsical fluids. The dissection of a limb, on which the operation for popliteal aneurism had been performed, by Mr. Astley Cooper, and a case of premature puberty in a female by Dr. Wall, are not the least interesting among these papers.

Whenever the opportunity has arisen, we have been anxious to recommend an attention to the topographical facts of the British islands, as far as they have any relation to the practice of physic, natural history, or any department of science, directly or indirectly connected with the medical art. The present interval enables us to state, if not a direct discovery, a full ascertainment and public relation of a topographical circumstance of considerable importance.

A few years since, Mr. Waterworth, an apothecary of Newport in the Isle of Wight, while employed in exploring the mineral springs of that island, discovered a mineral water at Sandrocks. It appears from a Report now published by Dr. William Lempriere, that the spring at Sandrocks is an aluminous chalybeate, more strongly impregnated with iron than any other water hitherto known. A

pint pint contains $\frac{3}{10}$ of a cubic inch of carbonic acid, about 41 grains of sulphate of iron, 31 grains of sulphate of alumina, 10 grains of sulphate of lime, 3 grains of sulphate of magnesia, 16 grains of sulphate of soda, 4 grains of muriate of soda, and a very trifling portion of silica. Its medicinal properties depend on the iron and alum which it holds in solution. Dr. Lempriere, who is physician to the military depôt in the Isle of Wight, in many cases of debility which the Walcheren fever left him to contend with, found this water an admirable remedy. "Sixty patients were selected in whom the paroxisms of ague had been suspended, but who were left in a state of great debility. In about three weeks, thirty-six of these patients were restored to health; eight were obliged to omit the water, in consequence either of relapses, or the supervention of other diseases; and sixteen continued the water whilst they were in a progressive state of recovery." Dr. Saunders attests, from his own experience, its efficacy in cases of uterine hæmorrhage, excessive discharges of fluor albus, and in incipient cases of diseased uterus. From the strength of the impregnation, and from the known properties of the impregnating materials, may be anticipated the efficacy of this water.

The following relation (vide Medico-Chirurg. Trans.) of an extraordinary and fatal disease, occurring in a remote part of the island, and arising from some local though *unknown* cause, is a pregnant instance of the value of recording medico-topographical facts. And, though, in this instance, there is not even a conjecture of the cause of the malady, it is probable a minute inquiry might have discovered it to be connected with some local peculiarity.

The village of Blackaton is placed on one of the high declivities of Dartmoor, about eight miles from Ashburton: four families only live at it, and their general health, for many years, was good. On March 23d, 1807, Mr. Gervis, a medical practitioner, was called to visit a boy nine years old, in one of the four families of Blackaton. This family consisted of fourteen individuals, who all dined together on Sunday the 22d, in perfect health. At four in the afternoon of the 22d, this boy complained of pain in his head with slight slight rigors. During the night the pain in the head increased, and slight convulsions of the limbs were noticed. There was little thirst, and no increase of temperature. Early in the morning of the 23d a slight sickness supervened, and he soon became totally insensible, and much inclined to sleep. At ten o'clock A. M. the pupil was fully dilated, morbid heat of the skin, respiration free, tongue moist, countenance pale, pulse weak and 80. Late in the evening the symptoms the same. In the night he expressed fear on observing a candle in the room, but could not see his father, who was near him. Tuesday morning 24th he was sensible, pulse natural, but inflammation with violent pain about the ball of the (great) toe, extending to the ancle and knee. In a week he had recovered, and remained well.

His sister, a girl of 16, who was in perfect health the greater part of Monday the 23d, and sat up with her brother that night, was seized in the evening with rigors, succeeded by great heat, thirst, and restlessness, acute pain in the head, and slight soreness in the throat. At five o'clock, Tuesday morning, 24th, vomiting came on, and soon after a total privation of sight and sense. Between nine and ten o'clock the pupils were fully dilated, tunica albuginea completely suffused with blood, pulse not distinguishable, and she expired almost immediately. On her neck and breasts were large irregular purple spots; her teeth white, and tongue moist.

On Thursday evening the 26th (by mistake called the 20th), the father, who had attended his daughter to the grave, soon after ten o'clock was attacked with violent pain in one foot, which soon extended to the other, and from thence to the region of the kidney, where it remained so acute as to require his being supported in a sitting posture. At this time pulse 100 and weak, countenance remarkably pale, tongue moist, surface cold, a disposition to vomit, with slight pain in the head. At four P. M. the pain removed from the back to the stomach, and at eight he expired. There were no patechiæ, and his intellects was not disordered.

On the 29th of April, another son arose at six o'clock in the the morning in perfect health: at eight o'clock he had slight soreness in the throat, and pain in the head, with rigors, but no increase of heat. At four p. m. he grew sick, vomited, and immediately became insensible. At a quarter past eight, neither the action of the artery nor the stroke of the heart could be distinguished. Teeth and tongue moist, respiration free, pupil dilated. At half past eight he expired.

On the 8th of May, a boy eleven years old, who resided in the same village, but who had not seen either of the above persons, while under the disease, was attacked at eight p. m. with slight rigors, sore throat, pain in the head, and stiffness in the limbs; hot skin, flushed face, and much disposed to sleep. At four on the morning of the 9th he became sick, retched much, and almost immediately died. Many large irregular purple spots appeared on his neck and breast about an hour before he expired. His alvine discharges and breath were extremely fœtid, circumstances that did not occur in either of the other cases.

Here this dreadful visitation stopped. In no other families of the village did it appear, and we are informed that the neighboring parishes did not feel its destructive influence. The rapid fatality of its progress, (two patients died in 24 hours after the attack, and two in 13 hours,) the direct metestasis to the *sensorium commune*, the appearance of putrescence (as we yet call certain appearances in the living body, because they bear some slight outward resemblance to the putrefactive process in dead animal matter) in two instances, and its total absence in the other two, strongly excite curiosity, and should stimulate to inquiry. It does not seem from Mr. Gervis's communication, that any other professional person saw the cases, or that investigation extended beyond the 9th of May. If these remarks should excite a further inquiry into this impressive history, either by Mr. Gervis, or any other professional resident in the vicinity of Blackaton, we shall have a full reward for having made them.

The utility of the Bath, in all its varieties, is not now a question upon which any discussion need to be employed.

But

But the most commodious, extended, and efficient, means of applying this curative agent, is not only a subject for speculation, but for serious practical inquiry. How the first city on this earth, the *punctum saliens* of the world, may be supplied with this desideratum on a scale proportionate to the grandeur and vastness of its establishments, the myriads of its people, and its inexhaustible wealth, has been pointed out to the great council of the nation, in a "Report for establishing of Sea-water and other Baths in the Vicinity of London." Why that council, almost without inquiry, rejected a project approved by the philanthropist, the philosopher, and the physician, and demonstrated to be practicable by those whose profession it is to determine on such questions, cannot readily be comprehended. Notwithstanding, however, this retardation of the great view of a Society, the object of which is to bring the waters of the ocean, in all their purity, within reach of the meanest inhabitant of the British metropolis, there cannot be much doubt of its final accomplishment. As a project for perfecting the medical science of the empire in this particular branch, and as, perhaps, the grandest scheme that the art has ever witnessed, we are called upon to notice it; and to notice with approbation, because we cannot doubt the beneficial influence it would have on the health of a crowded city.

Beside the employment of literature on the science and the art of medicine as applied to theory and practice, or as a philosophical study of Nature, it has been exercised in forming the manners and conduct, and instructing in the institutes of professional morals. At the head of this class of writers stands the late Dr. John Gregory. His lectures on the duties and qualifications of a physician, are too well known to require recommendation, and too excellent to call for panegyric. In the same field many others have labored, if not with similar success, with a laudable industry, and a portion of beneficial effect. The latest of these, Mr. John Strang, a surgeon of the royal navy, in "Letters to a Student of Medicine on his commencing Practice, containing a comparison of the condition of naval, military, and private, practitioners,"

practitioners," has done a real service to the junior part of the profession, by the publication of precepts deduced from his personal knowledge and experience. The pamphlet is arranged in a series of letters, and the first is on the choice of the profession, or rather on the election of some particular department of it. The advice contained in it deserves the serious consideration of the student. It comes from a person who has himself mistaken his post, and, on that account, is more impressive.

Non ignara mali, miseris succurrere disco.

The second and third treat on the advantages and disadvantages of the naval service; the fourth is on the peculiarities of military service; the fifth contains remarks on private practice; the sixth and seventh go more into detail on the naval and military duties of the surgeon; and the eighth, and last, is on the disposal of time not professionally occupied.

A slight retrospective glance upon this Report will shew, we fear, that it possesses but little of that interest which arises from novelty. It is not, however, altogether barren of incidents that will be gratifying to those who desire the improvement and melioration of an art, the utility of which was never seriously called in question.

"People may dispute whether physic, on the whole, does more good or harm to mankind; just as they may dispute, whether the faculty of reason, considering how it is often perverted, really contributes to make human life more or less happy; whether a vigorous constitution and an independent fortune are blessings or curses to those who possess them; whether the arts and sciences in general have proved beneficial to mankind. Such questions afford opportunities for the display of eloquence, and for saying plausible and ingenious things; but still nobody doubts of the real and substantial advantages attending those acquisitions, if applied to their natural and proper uses."

*Princes'-street, Cavendish-square,* R.

*June 30th,* 1812.

